# Effect of gingival application of melatonin on alkaline and acid phosphatase, osteopontin and osteocalcin in patients with diabetes and periodontal disease

**DOI:** 10.4317/medoral.18832

**Published:** 2013-03-25

**Authors:** Antonio Cutando, Antonio López-Valverde, Rafel Gómez-de-Diego, Salvador Arias-Santiago, Joaquín de Vicente-Jiménez

**Affiliations:** 1 MD, DDS, PhD, Department of Special Care in Dentistry, School of Dentistry, University of Granada, Granada, Spain; 2 Department of Surgery, School of Dentistry, Faculty of Medicine, University of Salamanca, Salamanca, Spain; 3 Department of Odontology, Faculty of Health Sciences, University of Alfonso X, Villanueva de la Cañada, Madrid, Spain; 4 Department of Dermatology, San Cecilio University Hospital, Granada,Spain

## Abstract

Objectives: To assess the effect of topical application of melatonin to the gingiva on salivary fluid concentrations of acid phosphatase, alkaline phosphatase, osteopontin, and osteocalcin. Study Design: Cross-sectional study of 30 patients with diabetes and periodontal disease and 30 healthy subjects. Diabetic patients were treated with topical application of melatonin (1% orabase cream formula) once daily for 20 days and controls with a placebo formulation. Results: Before treatment with melatonin, diabetic patients showed significantly higher mean salivary levels of alkaline and acid phosphatase, osteopontin and osteocalcin than healthy subjects (P < 0.01). After treatment with melatonin, there was a statistically significant decrease of the gingival index (15.84± 10.3 vs 5.6 ± 5.1) and pocket depth (28.3 ± 19.5 vs 11.9 ± 9.0) (P < 0.001). Also, use of melatonin was associated with a significant reduction of the four biomarkers. Changes of salivary acid phosphatase and osteopontin correlated significantly with changes in the gingival index, whereas changes of alkaline phosphatase and osteopontin correlated significantly with changes in the pocket depth. Conclusions: Treatment with topical melatonin was associated with an improvement in the gingival index and pocket depth, a reduction in salivary concentrations of acid phosphatase, alkaline phosphatase, osteopontin and osteocalcin.

** Key words:**Melatonin, diabetes mellitus, alkaline phosphatase, acid phosphatase, osteopontin, osteocalcin.

## Introduction

Periodontal disease is a complex condition that may vary from gingivitis to extreme destruction of tooth-supporting tissue. Although bacterial infection and release of toxic bacterial products triggers a series of processes leading to damage of healthy tissues, a number of actions of the host’ immune response is also involved. However, the etiopathogenesis and pathophysiology of periodontal disease remains unclear.

A response of the organism to the periodontal infection includes the production of several intracellular enzymes (1). Alkaline phosphatase and acid phosphatase are intracellular enzymes present in most tissues and organs, particularly in bones. Some studies have shown a remarkably increase in the activity of these enzymes in periodontitis and a reduction after periodontal therapy (2,3). Also, salivary alkaline phosphatase levels may be useful as a potential bone turnover marker to establish the diagnosis and prognosis of periodontal disease (4). On the other hand, periodontal tissue destruction via osteoclastic action results in the sequestration of bone specific matrix proteins, like telopeptides type I collagen (5), osteocalcin (6), osteonectin (7), osteopontin (8) and bone phosphoprotein (9) in the gingival crevicular fluid, all of which have been positively associated with the progression of periodontal disease (10,11).

Melatonin, an indoleamine secreted by the pineal gland in a circadian manner, is a noteworthy free radical scavenger (12) and also plays an immunomodulatory role (13). Several studies have shown that melatonin stimulates the proliferation and synthesis of type I collagen and promotes bone formation (14,15). Melatonin may have implications in diseases of the oral cavity, limiting tissue damage that is a result of free radicals, stimulating the immune response and reducing the progressive loss of alveolar bone (16,17). Previous studies of our group have shown the periodontal protective role of melatonin (18,19).

In order to assess the usefulness of melatonin application as a potential therapeutic strategy in periodontal processes, a cross-sectional study was conducted, the objective of which was to determine the effect of topical application of melatonin on salivary concentrations of alkaline phosphatase, acid phosphatase, osteocalcin and osteopontin in patients with diabetes and periodontal disease and in a control group of healthy subjects. The effect of topical melatonin on changes in clinical parameters, including the gingival index and the pocket depth was also assessed.

## Material and Methods

-Participants

The study was carried out at the Health Center of Pinos Puente (Granada, Spain). A total of 30 healthy individuals of both sexes (20 men, 18 women), aged 31 to 68 years (mean ± standard deviation, 47.0 ± 10.3 yrs) without periodontal disease and 30 patients with diabetes and periodontal disease of both sexes (14 men, 16 women) aged 24 to 58 years (mean 43.1 ± 12.4 yrs) were included in the study. There were 17 patients with type I diabetes and 13 with type II diabetes. All patients were free of medication (except for the treatment of diabetes). The type of periodontal disease was not an inclusion criterion for the study, although most patients presented advanced periodontitis. Other exclusion criteria included current use of bisphosphonates, oral contraceptives, antibiotic treatment in the previous 6 months, and having received (within the last 6 months) or being treated for diseases of the oral cavity. All healthy subjects were in good general health with no history of systemic disease or clinical signs of periodontal disease.

Participants were fully informed about the study and gave written informed consent. The study protocol was approved by the Ethics Committee of the Faculty of Odontology, University of Granada, and the code of Ethics of the World Medical Association was observed.

All study participants (patients and control) underwent an oral examination, including medical, dental, and caries assessments. The same dentist performed all examinations. Periodontograms were performed using the Florida Probe® handpiece (computerized periodontal probing system). The gingival index, bleeding on probing, and probing depth (20) were registered in patients with diabetes and in healthy subjects. Thereafter, patients with diabetes were treated with topical application of melatonin (1% orabase cream formula) both in the upper and lower dental arches on the surfaces of the attached gingiva for 20 days. Healthy subjects were treated with a placebo orabase cream. Melatonin cream (or placebo) was applied daily at night after routine oral hygiene. All participants were instructed how to use the orabase cream; it was recommended to apply the amount that fits in a regular toothbrush for adults per dental arch. Conventional periodontal treatment prior or during the study was not allowed. Oral examination was also performed at the end of treatment.

-Saliva collection

Before and after treatment, salivary samples from diabetic patients and healthy controls were collected. Patients and controls came to the School of Dentistry of the University of Granada at 09:00 AM after 12-h overnight fast. After 15 min of rest, a sample of saliva was obtained from each individual. In order to stimulate saliva production, the participants chewed a piece of paraffin wax for 7 min. Saliva produced during the first 2 min was discarded. Then, saliva was collected during the following 5 min, to avoid any possible contamination. The patients chewed the paraffin during the time of saliva collection. Samples of collected saliva were centrifuged at 3,000 g, 4ºC for 15 min, and then the clear supernatant was frozen at -80ºC until assays were performed.

-Analytical determinations 

The activity of acid and alkaline phosphatase in saliva was determined spectrometrically by the International Federation of Clinical Chemistry (IFCC) method on the Hitachi 911 Automatic Analyzer. Osteocalcin measurements in saliva were made using an electrochemiluminescence technique (Immulite 2000, Diagnostic Products Corporation, Los Angeles, CA, USA). The concentration of osteopontin was determined by sandwich-type of a human osteopontin enzyme immunometric assay (EIA) kit (TiterZyme, Assay Designs, Ann Arbor, MI, USA) according to the manufacturer’s instructions.

-Statistical analyses

Quantitative variables are expressed as the mean ± standard deviation (SD). The paired Student´s t test was used for the comparison of the gingival index and probing depth before and after topical application of melatonin among patients with periodontal diabetic disease, and the t test for independent samples for the comparison of salivary levels of acid phosphatase, alkaline phosphatase, osteocalcin and osteopontin between the groups of diabetic patients and healthy subjects. The relationship between the gingival index and probing depth with acid phosphatase, alkaline phosphatase, osteocalcin and osteopontin levels in saliva was assessed with the Pearson´s correlation coefficient. Statistical significance was set at P < 0.05. The Statistical Package for the Social Sciences (SPSS) (version 11.0) was used for the analysis of data.

## Results

**Figure 1:**
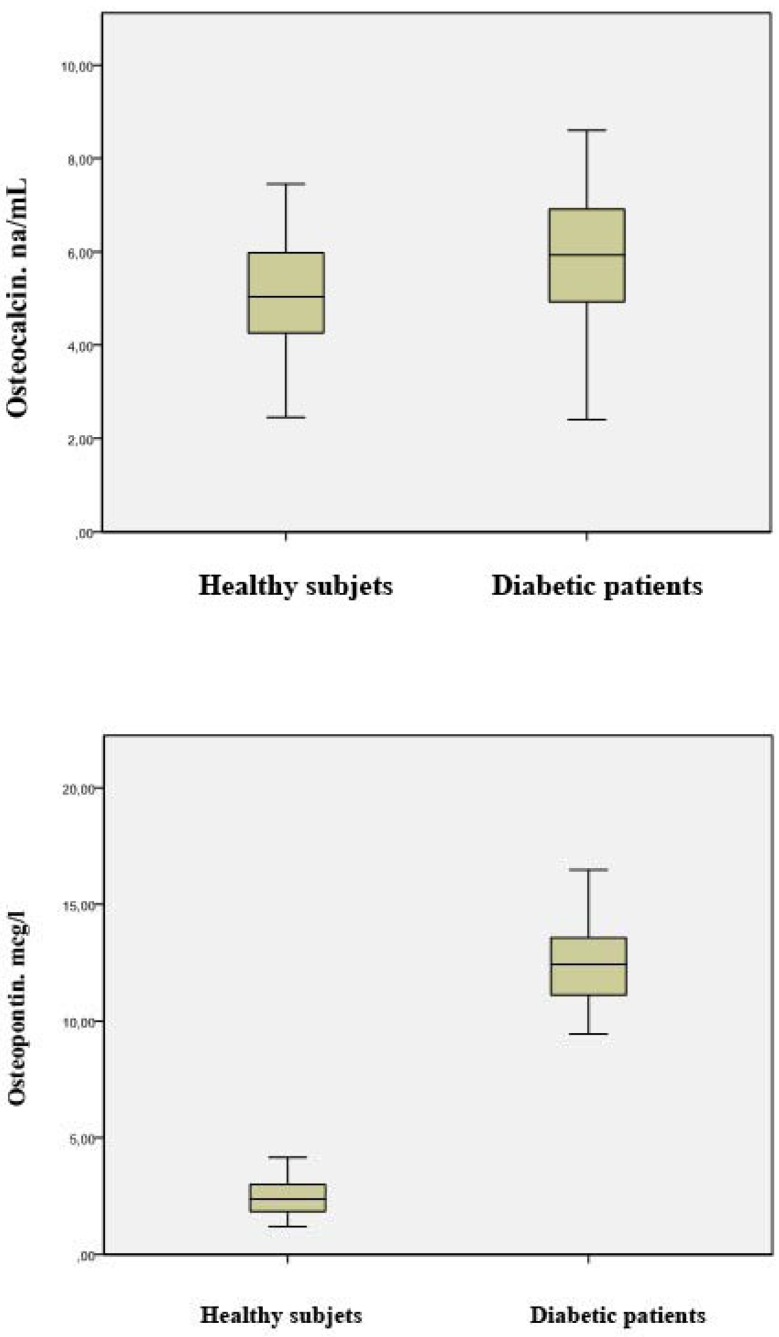
a) Box plot of the differences in salivary osteocalcin levels between healthy subjects and diabetic patients before topical application of melatonin. b) Box plot of the differences in salivary osteopontin between healthy subjects and diabetic patients before topical application of melatonin.

**Figure 2:**
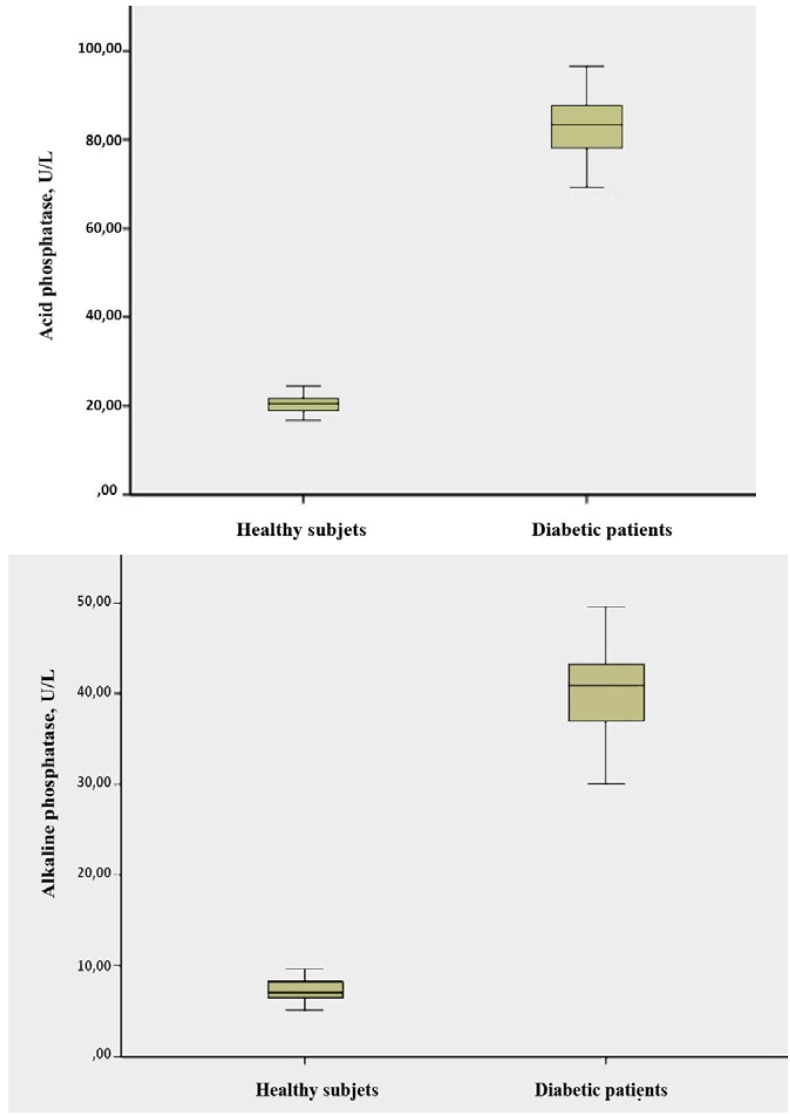
a) Box plot of the differences in salivary alkaline phosphatase levels between healthy subjects and diabetic patients before topical application of melatonin. b) Box plot of the differences in salivary acid phosphatase levels between healthy subjects and diabetic patients before topical application of melatonin.

The comparison of salivary levels of acid and alkaline phosphatases, osteocalcin and osteopontin between diabetic patients and healthy controls before treatment with melatonin is shown in  Table 1. Patients with diabetes had significantly higher mean levels of salivary acid phosphatase (83.08 ± 6.85 vs 20.55 ± 1.99 U/L) (P < 0.001), alkaline phosphatase (40.51 ± 4.83 vs 7.34 ± 1.28 U/L) (P < 0.001), osteocalcin (5.83 ± 1.41 vs 4.97 ± 1.35 ng/mL) (P = 0.020) and osteopontin (12.49 ± 1.78 vs 2.44 ± 0.80 µg/mL) (P < 0.001) than healthy subjects (Figs. 1,2).

**Table 1:**
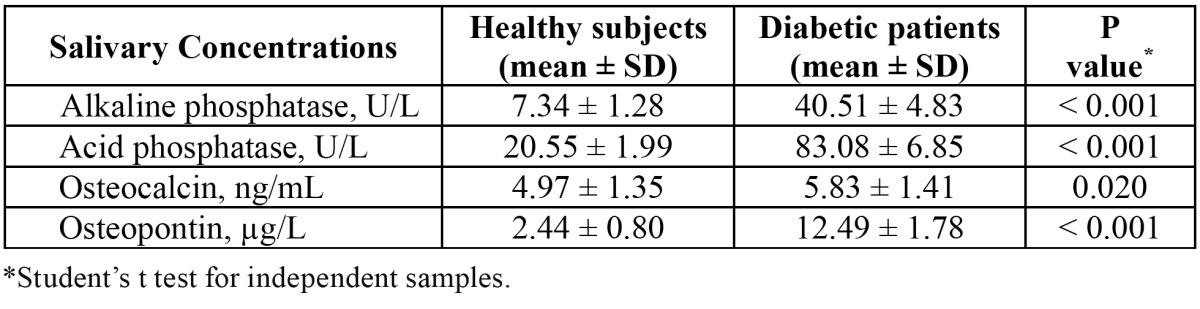
Comparison of patients with diabetes and healthy controls before topical gingival treatment with melatonin.

After the application of topical melatonin, there was a statistically significant reduction of the gingival index (15.84 ± 10.26 vs 5.59 ± 4.08), pocket depth (28.29 ± 19.48 vs 11.9 ± 9.01) and salivary levels of alkaline phosphatase (40.51 ± 4.83 vs 26.88 ± 4.03 ng/mL) (P < 0.001), acid phosphatase (83.08 ± 6.85 vs 43.2 ± 5.52) (P < 0.001), osteocalcin (5.83 ± 1.41 vs 5.78 ± 1.39 ng/mL) (P = 0.028) and osteopontin (12.49 ± 1.78 vs 8.34 ± 1.45 µg/mL) (P < 0.001) ( Table 2).

**Table 2:**
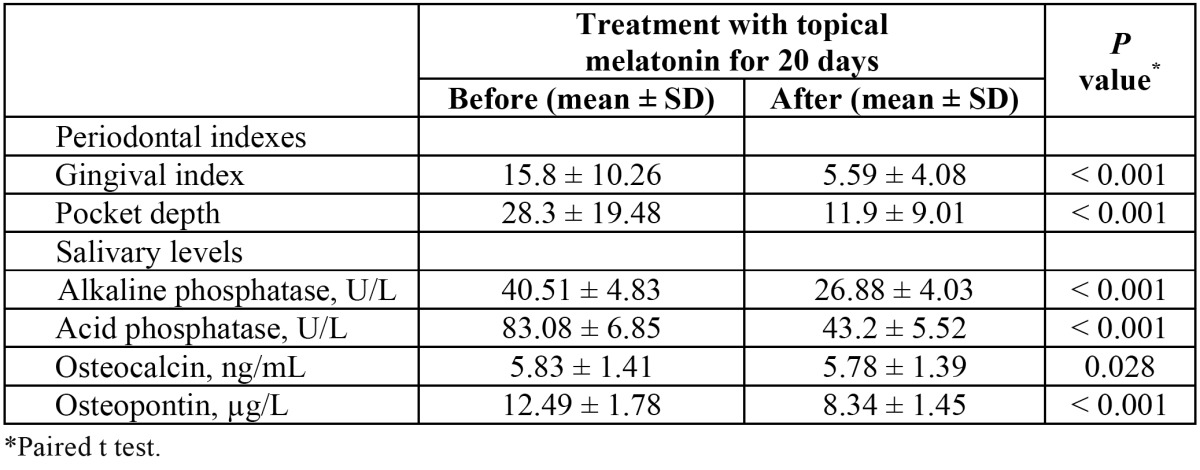
Comparison of gingival index, pocket depth and salivary levels of alkaline phosphatase, acid phosphatase, osteocalcin and osteopontin before and after topical application of melatonin.

There was also a significant association between changes in the gingival index before and after treatment with melatonin and changes in salivary levels of acid phosphatase (r = 0.499, P = 0.005) and osteopontin (r = 0.578, P = 0.001). Changes in the pocket depth correlated with changes in salivary levels of alkaline phosphatase (r = 0.366, P = 0.047), acid phosphatase (r = 0.443, P = 0.014) and osteopontin (r = 0.739, P = 0.001) ( Table 3).

**Table 3:**
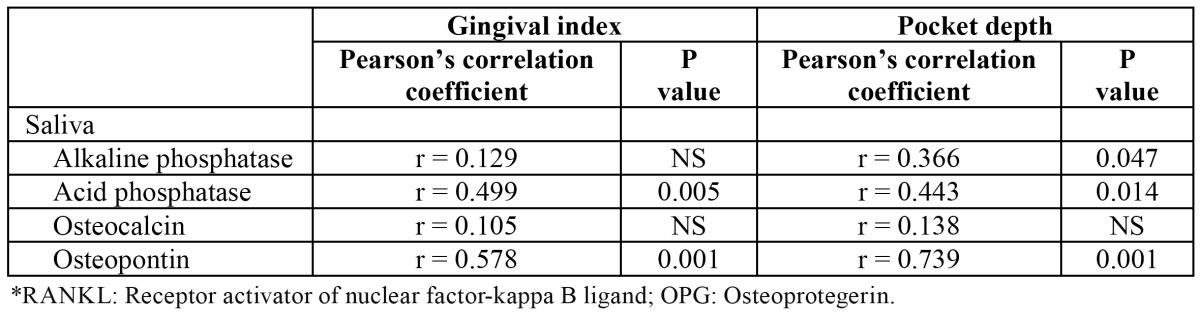
Relationship between changes of gingival index and pocket depth and changes of salivary RANKL*, salivary OPG*, salivary melatonin and plasma melatonin levels before and after treatment with melatonin.

## Discussion

It has been shown that the concentration in saliva of some intracellular enzymes, such as 

alkaline phosphatase and acid phosphatase are elevated in patients with periodontal disease (20); thus, increased salivary levels of these enzymes are regarded as valuable biochemical markers of alveolar bone destruction in active periodontal disease (2). Accordingly, a decrease in the salivary fluid levels of these enzymes would indicate lower alveolar bone destruction (21,22).

Osteocalcin, a protein found in the extracellular matrix of bone and dentin and involved in regulating mineralization in the bones and teeth, is currently described as a specific marker of osteoblast function (23). Moreover, different studies have evaluated os-teocalcin levels in crevicular fluid as a potential marker of abnormal bone turnover in periodontal disease progression (24). Similarly, osteopontin levels in crevicular fluid have been considered a marker of alveolar bone destruction (25).

The objective of this study was to evaluate the effect of topical application of melatonin to the gingiva on clinical indexes of periodontitis and salivary levels of phosphatases and osteocalcin and osteopontin as markers of alveolar bone status. The study population included a group of patients with diabetes and periodontal disease and a group of healthy subjects. The association between diabetes and periodontal diseases is well-established. Diabetes is a risk factor for periodontal disease, with diabetic patients exhibiting an increased prevalence, extent and severity of gingivitis and periodontitis compared to healthy adults (26). On the other hand, sialochemistry has been increasingly used for the assessment of periodontal disease indicators (27). We found that treatment with melatonin was associated with improvement in the gingival index and pocket depth, and a rise in salivary concentrations of acid and alkaline phosphatases, osteocalcin and osteopontin. These findings suggest that melatonin may have a favourable effect in slowing osteoclastogenesis, improving the quality of alveolar bone and preventing the progression of periodontal disease. The protective role of melatonin against periodontal disease has been also demonstrated in other studies (28,29).

The present findings, however, should be interpreted taking into account some limitations of the study, in particular the small sample size. However, these encouraging preliminary findings in a reduced number of diabetic patients and healthy controls will hopefully stimulate further research to elucidate the potential usefulness of treatment with melatonin to improve periodontal disease.

In summary, treatment with melatonin on the gingiva was associated with an improvement in the gingival index and pocket depth, and an increase in salivary concentrations of alkaline phosphatase, acid phosphatase, osteocalcin and osteopontin. This indicates that melatonin may exert a beneficial effect on decreasing periodontitis and in slowing osteoclastogenesis, improving the quality of alveolar bone and preventing the progression of periodontal disease.

## References

[1] Nakashima K, Giannopoulou C, Andersen E, Roehrich N, Brochut P, Dubrez B (1996). A longitudinal study of various crevicular fluid components as markers of periodontal disease activity. J Clin Periodontol.

[2] Dabra S, Singh P (2012). Evaluating the levels of salivary alkaline and acid phosphatase activities as biochemical markers for periodontal disease: A case series. Dent Res J (Isfahan).

[3] Perinetti G, Paolantonio M, Femminella B, Serra E, Spoto G (2008). Gingival crevicular fluid alkaline phosphatase activity reflects periodontal healing/recurrent inflammation phases in chronic periodontitis patients. J Periodontol.

[4] Gibert P, Tramini P, Sieso V, Piva MT (2003). Alkaline phosphatase isozyme activity in serum from patients with chronic periodontitis. J Periodont Res.

[5] Talonpoika JT, HÃmÃlÃinen MM (1994). Type I collagen carboxyterminal telopeptide in human gingival crevicular fluid in different clinical conditions and after periodontal treatment. J Clin Periodontol.

[6] Lee AJ, Walsh TF, Hodges SJ, Rawlinson A (1999). Gingival crevicular fluid osteocalcin in adult periodontitis. J Clin Periodontol.

[7] Eley BM, Cox SW (1998). Advances in periodontal diagnosis. 10. Potential markers of bone resorption. Br Dent J.

[8] Kido J, Nakamura T, Asahara Y, Sawa T, Kohri K, Nagata T (2001). Osteopontin in gingival crevicular fluid. J Periodontal Res.

[9] Bowers MR, Fisher LW, Termine JD, Somerman MJ (1989). Connective tissue-associated proteins in crevicular fluid: potential markers for periodontal diseases. J Periodontol.

[10] Bullon P, Goberna B, Guerrero JM, Segura JJ, Perez-Cano R, Martinez-Sahuquillo A (2005). Serum, saliva, and gingival crevicular fluid osteocalcin: their relation to periodontal status and bone mineral density in postmenopausal women. J Periodontol.

[11] Sharma CG, Pradeep AR (2006). Gingival crevicular fluid osteopontin levels in periodontal health and disease. J Periodontol.

[12] Reiter RJ, Tan DX, Manchester LC, Qi W (2001). Biochemical reactivity of melatonin with reactive oxygen and nitrogen species: a review of the evidence. Cell Biochem Biophys.

[13] Radogna F, Diederich M, Ghibelli L (2010). Melatonin: a pleiotropic molecule regulating inflammation. Biochem Pharmacol.

[14] Cardinali DP, Ladizesky MG, Boggio V, Cutrera RA, Mautalen C (2003). Melatonin effects on bone: experimental facts and clinical perspectives. J Pineal Res.

[15] Park KH, Kang JW, Lee EM, Kim JS, Rhee YH, Kim M (2011). Melatonin promotes osteoblastic differentiation through the BMP/ERK/Wnt signaling pathways. J Pineal Res.

[16] Cutando A, GÃmez-Moreno G, Arana C, Acu-a-Castroviejo C, Reiter RJ (2007). Melatonin: potential functions in the oral cavity. J Periodontol.

[17] GÃmez-Moreno G, Guardia J, Ferrera MJ, Cutando A, Reiter RJ (2010). Melatonin in diseases of the oral cavity. Oral Dis.

[18] Cutando A, GÃmez-Moreno G, Villalba J, Ferrera MJ, Escames G, Acu-a-Castroviejo D (2003). Relationship between salivary melatonin levels and periodontal status in diabetic patients. J Pineal Res.

[19] Cutando A, Galindo P, GÃmez-Moreno G, Arana C, Bola-os J, Acu-a-Castroviejo D (2006). Relationship between salivary melatonin and severity of periodontal disease. J Periodontol.

[20] Daltaban O, Saygun I, Bal B, BaloÅ K, Serdar M (2006). Gingival crevicular fluid alkaline phosphatase levels in postmenopausal women: effects of phase I periodontal treatment. J Periodontol.

[21] Giannobile WV (1997). Crevicular fluid biomarkers of oral bone loss. Curr Opin Periodontol.

[22] Giannobile WV, Al-Shammari KF, Sarment DP (2003). Matrix molecules and growth factors as indicators of periodontal disease activity. Periodontol 2000.

[23] Bullon P, Chandler L, Segura Egea JJ, Perez Cano R, Martinez Sahuquillo A (2007). Osteocalcin in serum, saliva and gingival crevicular fluid: their relation with periodontal treatment outcome in postmenopausal women. Med Oral Patol Oral Cir Bucal.

[24] Becerik S, Afacan B, OztÃrk VÃ, Atmaca H, Emingil G (2011). Gingival crevicular fluid calprotectin, osteocalcin and cross-linked N-terminal telopeptid levels in health and different periodontal diseases. Dis Markers.

[25] Sharma CG, Pradeep AR (2007). Plasma and crevicular fluid osteopontin levels in periodontal health and disease. J Periodont Res.

[26] Lakschevitz F, Aboodi G, Tenenbaum H, Glogauer M (2011). Diabetes and periodontal diseases: interplay and links. Curr Diabetes Rev.

[27] Nomura Y, Tamaki Y, Tanaka T, Arakawa H, Tsurumoto A, Kirimura K (2006). Screening of periodontitis with salivary enzyme tests. J Oral Sci.

[28] GÃmez-Moreno G, Cutando-Soriano A, Arana C, Galindo P, Bola-os J, Acu-a-Castroviejo D (2007). Melatonin expression in periodontal disease. J Periodont Res.

[29] Srinath R, Acharya AB, Thakur SL (2010). Salivary and gingival crevicular fluid melatonin in periodontal health and disease. J Periodontol.

